# The Impact of Formal and Informal Volunteering Patterns on Health in Older Adults

**DOI:** 10.1093/geroni/igaf122.2392

**Published:** 2025-12-31

**Authors:** Huei-wern Shen, Peter Sun, Yi Wang

**Affiliations:** University of North Texas, Denton, Texas, United States; Bar-Ilan University, Ramat Gan, Tel Aviv, Israel; University of Iowa School of Social Work, Iowa City, Iowa, United States

## Abstract

In later life, many individuals participate in both formal and informal volunteering. While the health benefits of volunteering are well-documented, little is known about the impact of patterns of formal and informal volunteering on health outcomes among older adults. Using five waves of data (2010–2018) from the Health and Retirement Study, multichannel sequence analysis and cluster analysis were applied to identify volunteering patterns among 3,579 individuals aged 65 and older in 2010 who remained alive throughout the study period (17,895 observations). Four distinct clusters (C1-C4) were identified: (C1) Decreasing Informal Volunteering (37%), (C2) Steady Non-Involvement (31%), (C3) Decreasing High-Intensity Dual Involvement (17%), and (C4) Decreasing Low-Intensity Dual Involvement (15%). Then, controlling for sociodemographic characteristics, three regression models were used to explore the relationships between these clusters and three health indicators: cognitive function, self-rated health (SRH), and depressive symptoms (CESD). Compared to Cluster 2 (consistently non-volunteering older adults), those who engaged in volunteering (either formal or informal) during the study period (Clusters 1, 3, and 4) exhibited better cognitive function and fewer depressive symptoms, even as their involvement declined over time. However, only those in Cluster 3 (decreasing high-intensity dual involvement) reported significantly better self-rated health than the non-volunteering group. These findings highlight the lasting health benefits of volunteering in later life. Future research should explore the mechanisms underlying these relationships and investigate their relevance across diverse populations to provide further insights into the long-term effects of volunteering patterns on health.

